# The SMART Study, a Mobile Health and Citizen Science Methodological Platform for Active Living Surveillance, Integrated Knowledge Translation, and Policy Interventions: Longitudinal Study

**DOI:** 10.2196/publichealth.8953

**Published:** 2018-03-27

**Authors:** Tarun Reddy Katapally, Jasmin Bhawra, Scott T Leatherdale, Leah Ferguson, Justin Longo, Daniel Rainham, Richard Larouche, Nathaniel Osgood

**Affiliations:** ^1^ Johnson Shoyama Graduate School of Public Policy University of Regina Regina, SK Canada; ^2^ Johnson Shoyama Graduate School of Public Policy University of Saskatchewan Saskatoon, SK Canada; ^3^ College of Medicine Department of Community Health & Epidemiology University of Saskatchewan Saskatoon, SK Canada; ^4^ School of Public Health and Health Systems University of Waterloo Waterloo, ON Canada; ^5^ College of Kinesiology University of Saskatchewan Saskatoon, SK Canada; ^6^ Environmental Science Program Dalhousie University Halifax, NS Canada; ^7^ Faculty of Health Sciences University of Lethbridge Lethbridge, AB Canada; ^8^ Department of Computer Science College of Arts and Science University of Saskatchewan Saskatoon, SK Canada

**Keywords:** exercise, sedentary lifestyle, smartphone, ecological momentary assessments, epidemiological monitoring, translational medical research, health policy

## Abstract

**Background:**

Physical inactivity is the fourth leading cause of death worldwide, costing approximately US $67.5 billion per year to health care systems. To curb the physical inactivity pandemic, it is time to move beyond traditional approaches and engage citizens by repurposing sedentary behavior (SB)–enabling ubiquitous tools (eg, smartphones).

**Objective:**

The primary objective of the *Saskatchewan, let’s move and map our activity* (SMART) Study was to develop a mobile and citizen science methodological platform for active living surveillance, knowledge translation, and policy interventions. This methodology paper enumerates the SMART Study platform’s conceptualization, design, implementation, data collection procedures, analytical strategies, and potential for informing policy interventions.

**Methods:**

This longitudinal investigation was designed to engage participants (ie, citizen scientists) in Regina and Saskatoon, Saskatchewan, Canada, in four different seasons across 3 years. In spring 2017, pilot data collection was conducted, where 317 adult citizen scientists (≥18 years) were recruited in person and online. Citizen scientists used a custom-built smartphone app, Ethica (Ethica Data Services Inc), for 8 consecutive days to provide a complex series of objective and subjective data. Citizen scientists answered a succession of validated surveys that were assigned different smartphone triggering mechanisms (eg, user-triggered and schedule-triggered). The validated surveys captured physical activity (PA), SB, motivation, perception of outdoor and indoor environment, and eudaimonic well-being. Ecological momentary assessments were employed on each day to capture not only PA but also physical and social contexts along with barriers and facilitators of PA, as relayed by citizen scientists using geo-coded pictures and audio files. To obtain a comprehensive objective picture of participant location, motion, and compliance, 6 types of sensor-based (eg, global positioning system and accelerometer) data were surveilled for 8 days. Initial descriptive analyses were conducted using geo-coded photographs and audio files.

**Results:**

Pictures and audio files (ie, community voices) showed that the barriers and facilitators of active living included intrinsic or extrinsic motivations, social contexts, and outdoor or indoor environment, with pets and favorable urban design featuring as the predominant facilitators, and work-related screen time proving to be the primary barrier.

**Conclusions:**

The preliminary pilot results show the flexibility of the SMART Study surveillance platform in identifying and addressing limitations based on empirical evidence. The results also show the successful implementation of a platform that engages participants to catalyze policy interventions. Although SMART Study is currently geared toward surveillance, using the same platform, active living interventions could be remotely implemented. SMART Study is the first mobile, citizen science surveillance platform utilizing a rigorous, longitudinal, and mixed-methods investigation to temporally capture behavioral data for knowledge translation and policy interventions.

## Introduction

### Physical Inactivity and the Current Active Living Research Landscape

Physical inactivity is the fourth leading cause of death worldwide, costing approximately US $67.5 billion per year to health care systems globally [1,2]. According to the World Health Organization, rising physical inactivity and sedentary behavior (SB) can be attributed to the combined effects of urbanization and screen time–based, desk-bound lifestyles [3]. Responding to this phenomenon and the need for policy interventions to facilitate more active lifestyles, the interdisciplinary field of active living research (ALR) has gained prominence [4]. ALR envisions physical activity (PA) beyond exercise to include any movement in *free living* conditions in any environment (ie, home, school, work, and neighborhood). ALR focuses on both PA and SB, as these two behaviors are separate entities [5]. Accumulating high PA does not preclude one from accumulating high SB as well, and it is important to capture these distinct behaviors together because of their interrelationship [6].

Most ALR has been cross-sectional, generating correlational evidence that needs to be corroborated with objective, longitudinal studies [7-11]. Although surveys and other self-report measures have been validated for PA and SB, objective measurement tools (eg, accelerometers) that address limitations such as recall bias often pose logistical difficulties because of high costs and challenges with deployment [12,13]. Additionally, in measuring PA and SB, contextual information, which is imperative to understanding barriers and facilitators to active living, is often missing from objective measures. For instance, accelerometers do not capture *how* (type of activity), *where* (location), *why* (motivation), and *with whom* (social networks) PA and SB are accumulated.

The predominant emphasis of ALR has been on the outdoor environment, including urban design and neighborhood built environment [11,14]. However, individuals in Western nations spend much of their day being sedentary indoors. Although some consensus has been emerging in studying the influence of the indoor environment on SB, especially in home, occupational, and educational settings [15], thus far, progress in this area has been limited. Apart from Western lifestyles, another reason individuals spend prolonged periods of time indoors is because of adverse weather, with seasonal variations contributing to fluctuations in PA [16-23]. To date, ALR has focused on the influence of weather on PA patterns [19-23], ignoring the relationship between the outdoor or indoor physical environments, social environment, and weather [24,25].

In informing active living policies, it is imperative to understand pathways through which physical and social environments influence active living, and in turn, how active living influences downstream health outcomes (ie, weight status and cardiovascular disease risk) [26-29]. Moreover, given the role of individual motivation in engaging PA, it is also important to better understand how and if intrinsic motivation affects the direction of these pathways [30-33].

### Employing Citizen Science in Active Living Research

Capturing complex active living pathways, which includes physical, social, and individual determinants among populations, requires longitudinal surveillance, with consistent compliance from research participants. Citizen science is a participatory approach where participants, termed citizen scientists, actively engage in the research process from data collection to knowledge translation, thus improving the probability of longitudinal participant compliance [34]. Citizens’ involvement in cocreating knowledge enhances their role as constituents and advocates for the outputs of the research, which may provide impetus for decision makers to engage with researchers in implementing active living policies [35].

In recent years, the successful spread of virtual citizen science has been built upon the dispersal of Internet-connected computers around the globe [36]. The ubiquitous presence of smartphones allows researchers to leverage citizen-owned Internet-connected devices as mobile data collection tools. Currently, there are over 2.5 billion smartphone owners globally, and this number is projected to increase beyond 6 billion by the year 2020 [37]. These staggering numbers open up a pool of potential research participants never before available, each of them carrying their own technology capable of facilitating participation in research.

More importantly, the technology that powers these devices provides extraordinary research opportunities to overcome traditional constraints in terms of participant recruitment and retention, data collection and analysis, interventions, and knowledge translation. Smartphones have become a key part of day-to-day life and the primary one-stop communication device at home, at work, and on the go [38]. By utilizing sensors (eg, accelerometers and pedometers) embedded in smartphones and engaging with participants in their daily environments in real time (eg, via ecological momentary assessments, EMAs) [39,40], the current technology environment provides an opportunity to develop novel models of population health surveillance to address gaps in the current ALR landscape.

### Study Aims and Objectives

The objective of the *Saskatchewan, let’s move and map our activity* (SMART) Study is to develop and test a mobile citizen science platform for active living surveillance, knowledge translation, and policy interventions [41]. An important component of the platform is collaboration with key stakeholder groups (decision makers at multiple levels and citizen scientists) to ensure integrated knowledge translation and create opportunities for policy interventions in different settings.

SMART Study is a rigorous, longitudinal, and mixed-methods investigation employing citizen science in engaging participants via their smartphones. Participants are citizen scientists who play an important role in shaping data collection and driving knowledge translation. The key factor distinguishing SMART Study citizen scientists from participants in other studies is real-time engagement with researchers, where citizen scientists provide not only objective and subjective data with and without researcher-triggered prompts (self-motivated), but also their perceptions about data collection approaches (eg, timing of survey deployment).

The aim of this study was to understand how physical (eg, built environment and weather) and social contexts, as well as participant motivation, interact with each other to influence both PA and SB, and in turn, how PA and SB influences eudaimonic well-being. Eudaimonic well-being is a positive and holistic indicator of mental health, which is characterized as fulfilling or realizing one’s true potential [42-46]. The purpose of measuring eudaimonic well-being is to connect active living with a positive mental health outcome.

Through citizen engagement and collaborations with decision makers at multiple levels (provincial, local jurisdictional, community, and institutional levels), SMART Study’s ultimate purpose is to facilitate integrated knowledge translation and influence active living policies. This is a methodology paper that enumerates the SMART Study platform’s conceptualization, design, implementation, data collection procedures, analytical strategies, and potential for informing policy interventions. In doing so, preliminary and aggregated results and knowledge translation efforts from pilot data collection are discussed to depict the flexibility of the methodological platform to adapt longitudinally.

## Methods

### Study Overview

SMART Study is a prospective cohort study designed to obtain longitudinal data from a convenience sample of adults (≥18 years) from the two largest urban centers in Saskatchewan, Canada (Regina and Saskatoon). Out of Saskatchewan’s population of 1,033,381, Saskatoon and Regina census metropolitan areas account for 260,600 and 210,556 people, respectively [47]. The longitudinal data collection is scheduled in different seasons (winter, spring, summer, and autumn), with each seasonal cycle consisting of 8 consecutive days of data collection. After scrutinizing the historical weather data for seasonality in Regina and Saskatoon [48-51], January or February, April or May, July or August, and October or November have been selected as the months to include for seasonal data collection cycles to capture data in all seasons and account for the influence of seasonality on active living.

### Pilot Recruitment and Data Collection Strategy

To track longitudinal behavior patterns and their influence on health outcomes within the context of seasonal variations, all types of data (PA, SB, perception of environment, individual motivation, and eudaimonic well-being) will be collected in different seasons. The pilot data collection cycle (1st cycle), which was scheduled between April 1 and May 31, 2017, resulted in the recruitment of 317 citizen scientists. Our goal is to recruit and retain 1000 citizen scientists throughout the 3-year study period. Ethics approval was obtained from the universities of Regina and Saskatchewan through a synchronized review protocol.

**Figure 1:**
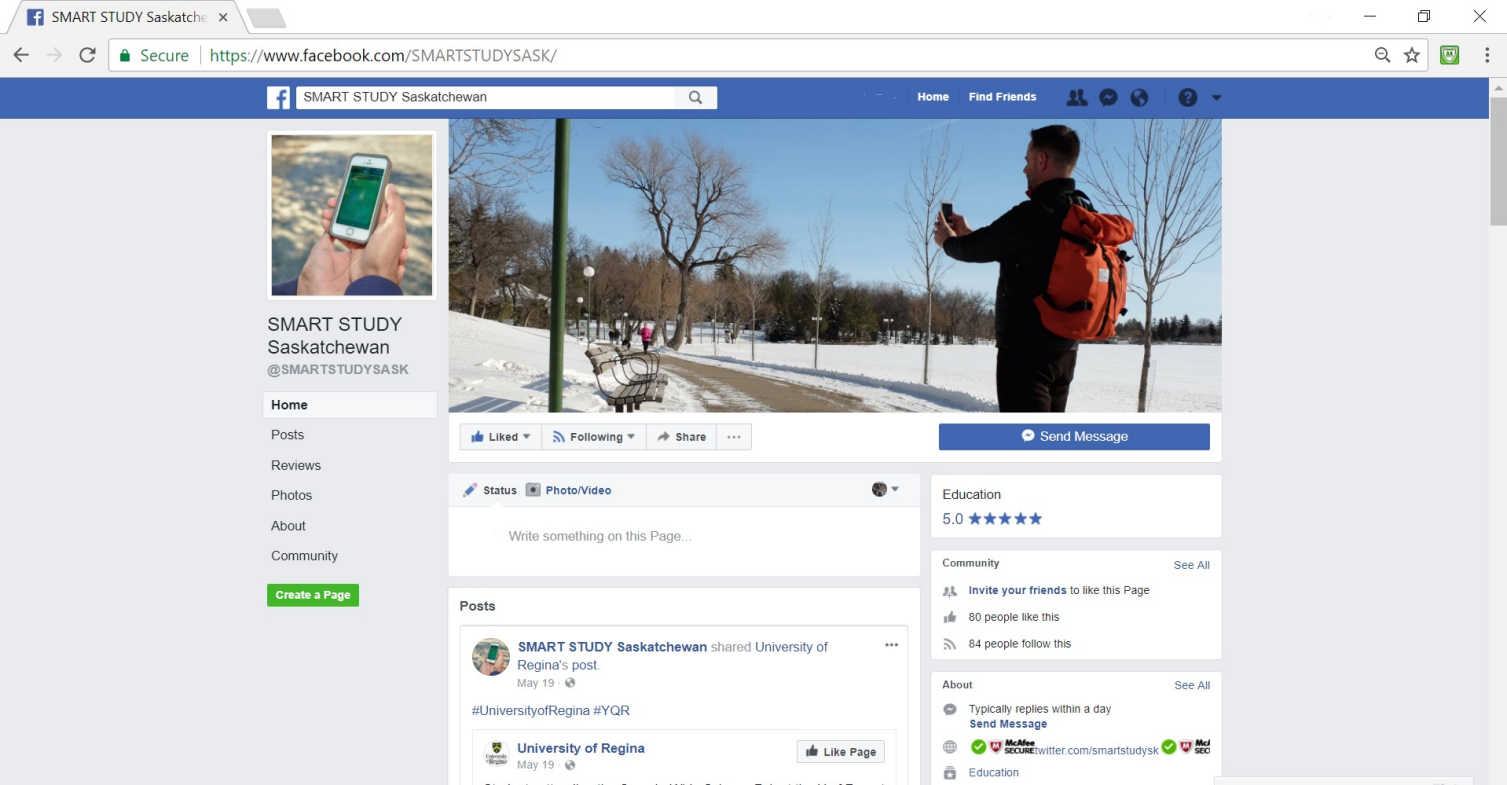
*Saskatchewan, let’s move and map our activity* (SMART) Study: Social media campaign.

**Figure 2:**
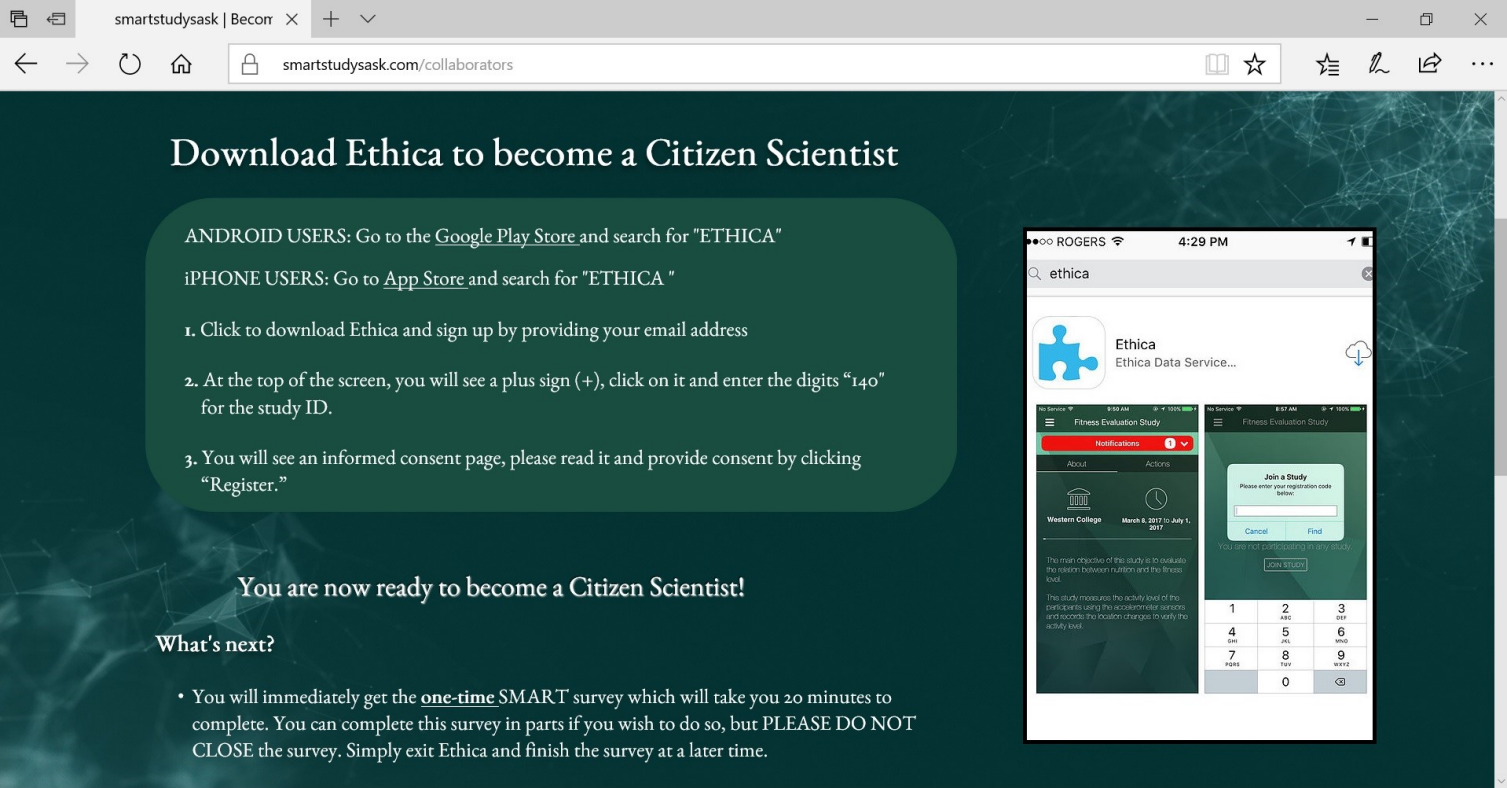
Online instructions to become a citizen scientist.

Aided by a social media campaign that raised awareness of the project in the two jurisdictions (Figure 1), citizen scientists were recruited in person from community centers located in different areas in each city and the universities of Regina and Saskatchewan to ensure recruitment of a representative sample. During in-person recruitment, potential citizen scientists were guided in downloading Ethica (Ethica Data Services Inc), an epidemiological mobile phone app specifically adapted for the SMART Study. Citizen scientists had the option to download the app using both Android phones and iPhones through the Google Play Store and Apple App Store. The social media campaign also directed interested participants to visit the study website to learn more about the study and to become citizen scientists by downloading the study app (Figure 2).

**Figure 3:**
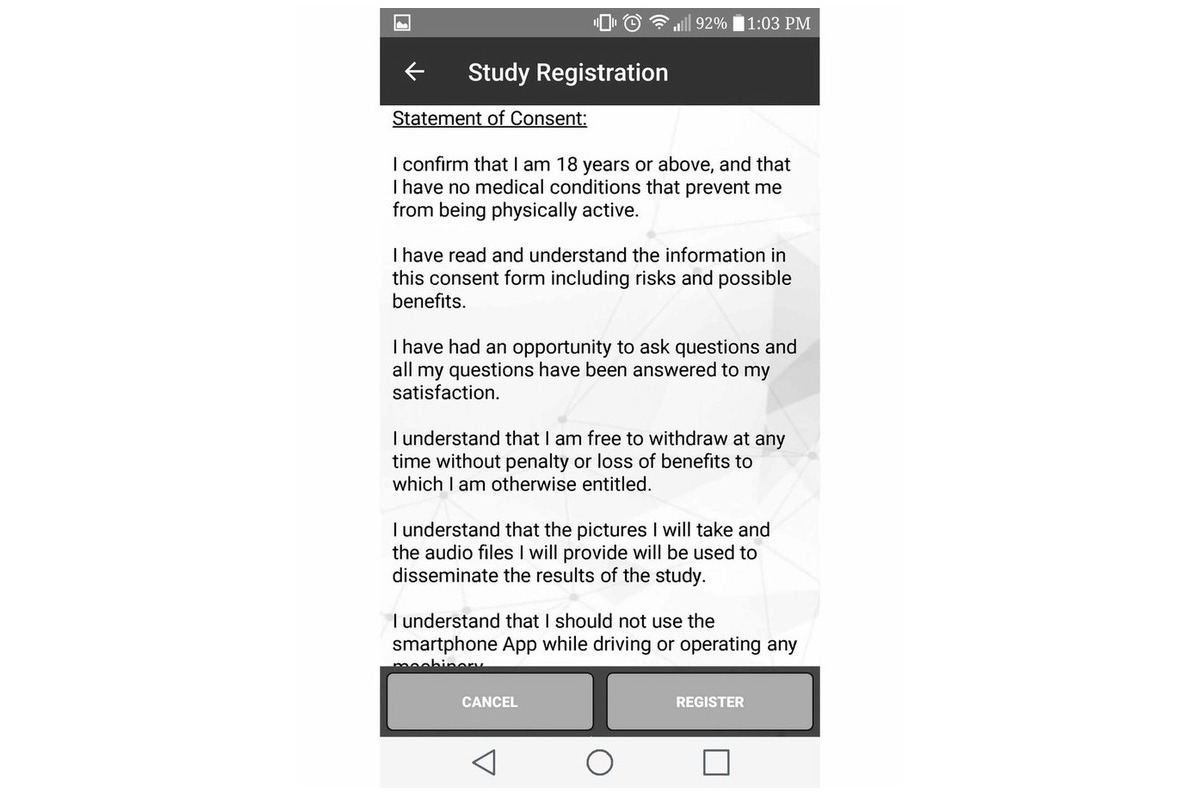
Informed consent provided via smartphone app.

**Figure 4:**
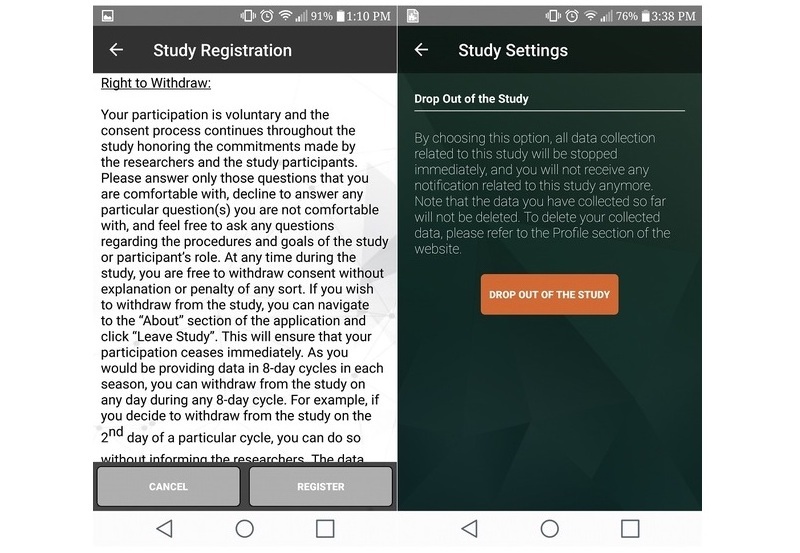
Study dropout option in the smartphone app.

All potential citizen scientists, whether they were recruited in person or online through social media, were required to complete an informed consent process through the app (Figure 3) and confirm their age (≥18 years) before being recruited. Citizen scientists were informed that the initial data collection period would last 8 consecutive days from the day they downloaded the app, followed by similar data collection cycles in different seasons, where citizen scientists would have to provide informed consent each time they participated. With the exception of demographic and historical PA data, all data will be collected in 8-day cycles in different seasons over 3 years from the same citizen scientists. Citizen scientists were also informed that they were free to withdraw from the study without explanation or penalty anytime during the 8-day data collection period. Clear instructions were provided regarding study withdrawal within the app (Figure 4). Citizen scientists were informed not to change their usual mobile phone–carrying habits so as to capture objective mobile phone sensor data in free-living conditions.

### Data Collection Tools

The SMART Study adapted the Ethica app by modifying features ranging from processes of informed consent and participant recruitment to survey development, deployment and triggering mechanisms, and selection of appropriate sensors that enable real-time data collection. Ethica is being used to collect a wide variety of valid and reliable health data in different geographic regions (high-density urban and rural regions) and in diverse populations such as university students and lower-income communities across North America [52-54], Australia, and Europe. It allows both subjective (via surveys) and objective (via smartphone sensors) data collection. Beyond typical objective and subjective data, Ethica can also enable capture of contextual perceptions via smartphone camera and audio function, as well as deployment of EMAs.

### Sensor-Based Objective Data

**Figure 5:**
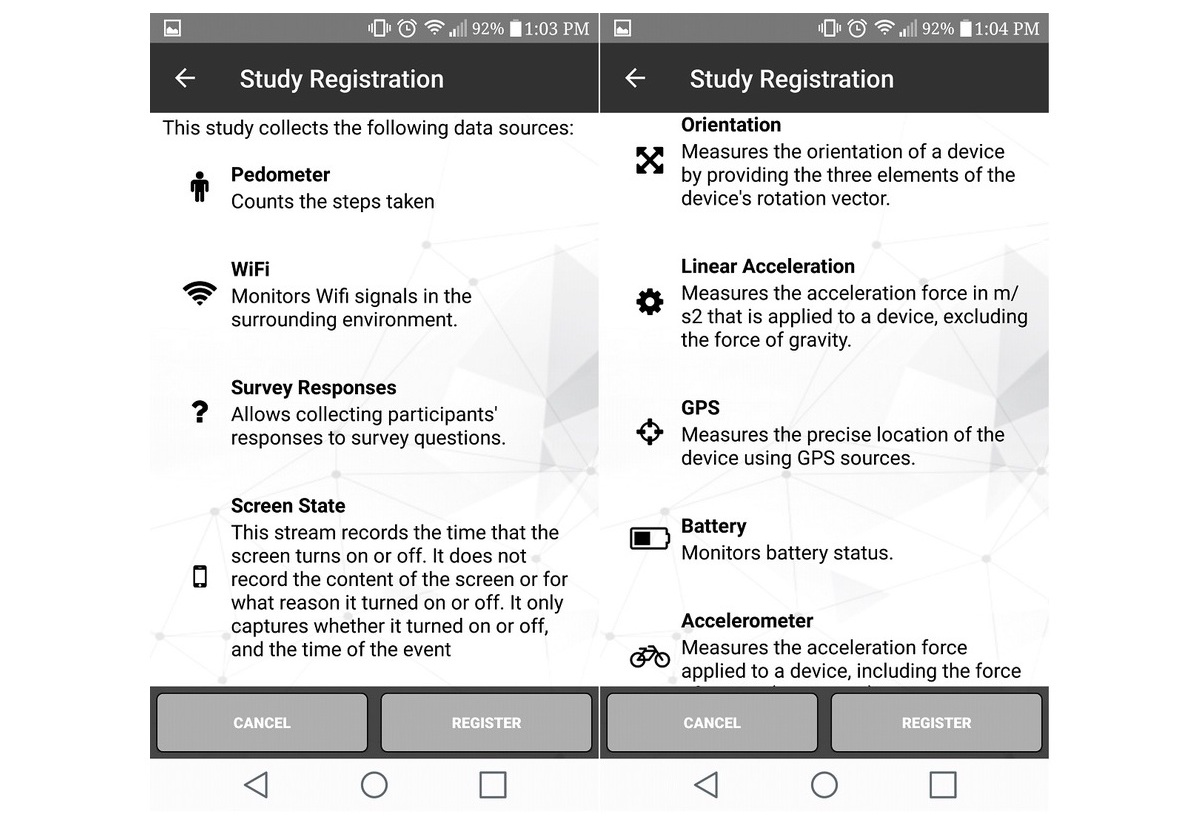
*Saskatchewan, let’s move and map our activity* (SMART) Study sensors.

Six smartphone sensors were used to obtain the following time-stamped objective data for 8 consecutive days: (1) accelerometer (activity intensity and frequency-25 second epochs), (2) pedometer (step counts-1 record/step), (3) screen state (smartphone screen time-1 record/event, ie, screen turned on or off), (4) global positioning systems (GPS; location-1 minute of GPS data/5 min), (5) Wi-Fi (proxy for indoor location or usage—proximity to all Wi-Fi access points once every 5 min), and (6) battery (smartphone usage or compliance-1 record/5 min; Figure 5).

### SMART Survey

**Figure 6:**
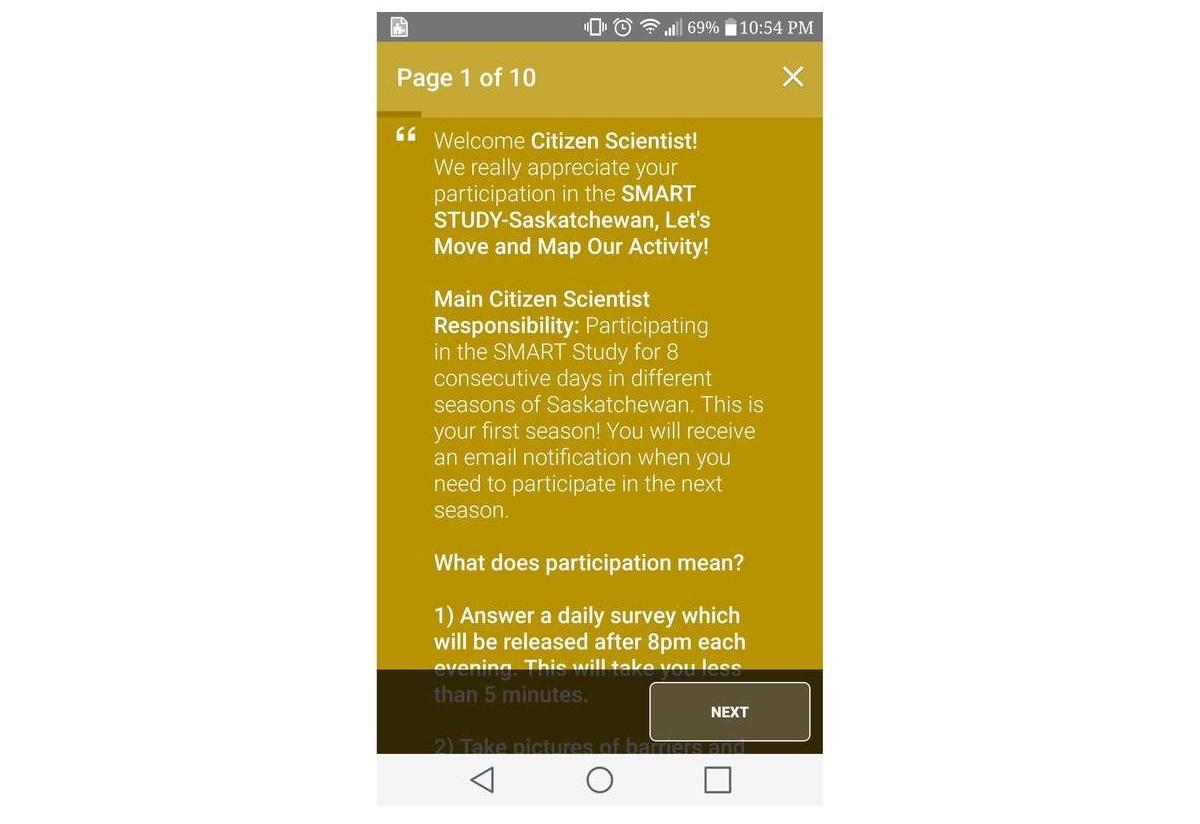
*Saskatchewan, let’s move and map our activity* (SMART) survey.

SMART survey (Figure 6) is the primary 209-item integrated questionnaire that combines validated self-report surveys to record PA, SB, motivation, eudaimonic well-being, and perception of outdoor and indoor environment. PA data were collected using the 27-item International PA Questionnaire (IPAQ), which measures PA domains [55,56]. The 9-item SB Questionnaire was adapted to measure different types of screen time and non-screen time–based SB on weekdays and weekends [57]. To capture the complexity of screen time accumulation, citizen scientists were given the option of recording common screen time behaviors (television, Internet surfing, and video games) over a range of digital devices (computer, laptop, tablet, smartphone, etc). Individual motivation and eudaimonic well-being were collected using the 16-item Motivation for PA Questionnaire [58] and the 21-item Questionnaire for Eudaimonic Well-being [59].

Perception of outdoor built environment was measured by using the 17-item PA Neighborhood Environment Survey [60]. Utilizing evidence from emerging studies [61], an indoor built environment survey was developed and used to capture participants’ perception of the indoor environment at home, work, and any fitness center associated with the participant [59]. Apart from these surveys, citizen scientists were also asked to report demographic and historical PA data (age, gender, income, education, employment, activity levels in adolescence in different seasons, etc), height (in feet or inches or cm), and weight (in pounds or kg) to calculate body mass index and perception of their health (self-rated health).

### Ecological Momentary Assessments

**Figure 7:**
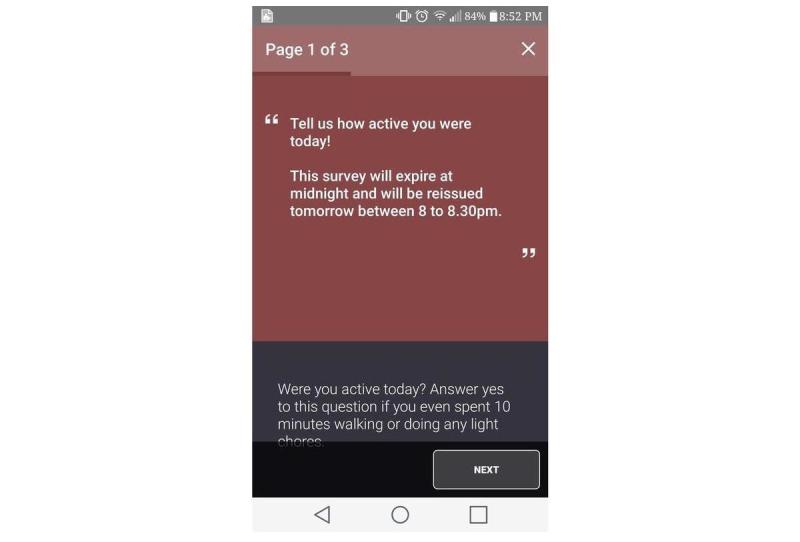
Ecological momentary assessment 1.

**Figure 8:**
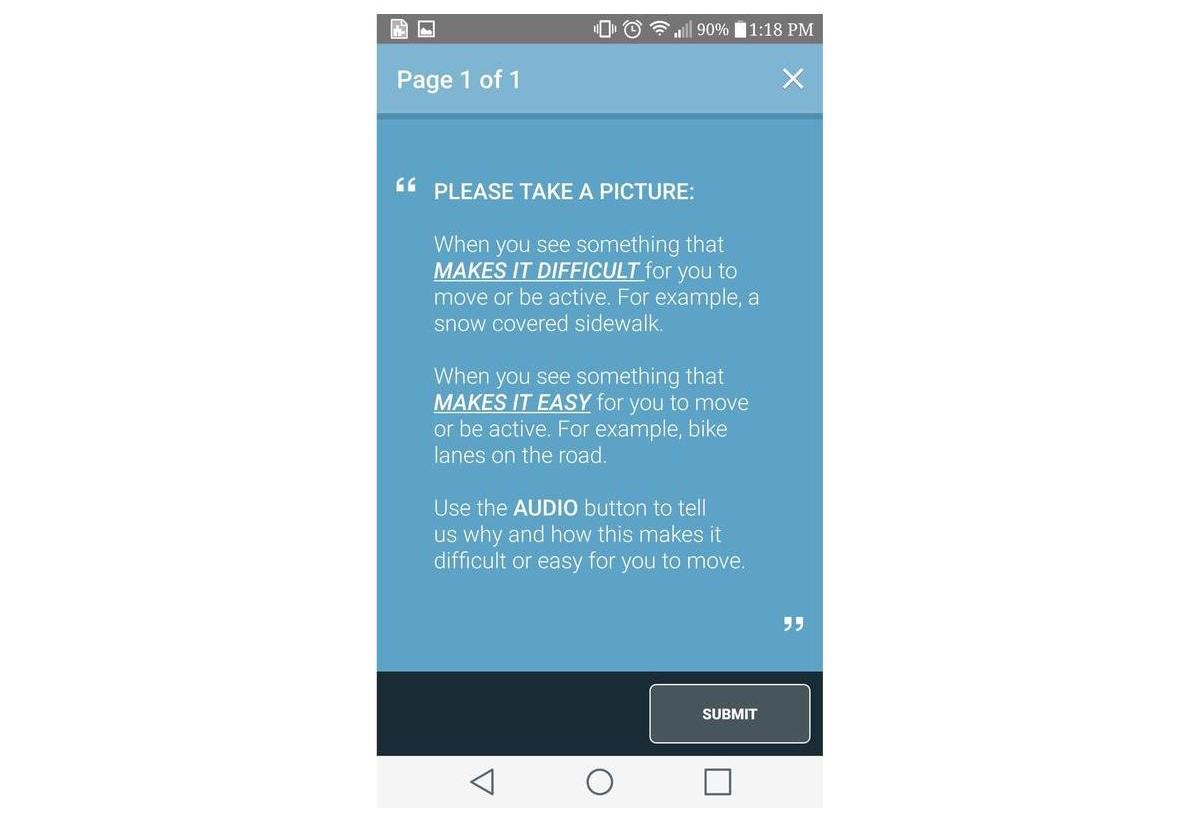
Ecological momentary assessment 2.

Two EMAs were deployed in the first cycle. The first EMA recorded daily PA types or intensities along with the physical and social contexts within which these activities were accumulated (Figure 7). The second EMA (Figure 8) captured barriers and facilitators of active living by asking citizen scientists to randomly take pictures of their outdoor or indoor environment. After citizen scientists took pictures, they were prompted to describe the pictures using an audio recording to explain how they perceived their immediate environment to be a barrier or facilitator of active living. These pictures and audio files were automatically geo-coded to the exact latitude-longitude location as measured via smartphone–based location services.

### Paradata

Objective paradata, which are essentially the by-products of research, are automatically generated using Ethica [62,63]. Paradata can be classified as device-specific or participant-specific. Examples of device-specific paradata include battery level and proximity to Wi-Fi networks or Bluetooth devices. Examples of participant-specific paradata include number of nudges it takes for participants to respond to a survey and the time it takes for participants to complete surveys (as a whole or questions therein).

Apart from the objective sensor-based paradata, three separate feedback surveys were triggered to obtain important paradata regarding EMAs and the citizen scientists’ response to SMART Study itself (ie, if participating in SMART Study changed their activity behavior, and if yes, how and why?). These surveys could provide evidence to inform future methodological decisions such as survey triggering and expiry mechanisms.

### Survey Triggering and Expiry Mechanisms

Over the 8-day data collection period, various triggering mechanisms (time vs user-triggered) were used to deploy different surveys. Time-triggers (ie, prompts) are controlled, preselected time points on specific days, and user-triggers are citizen scientist–controlled and can be randomly triggered by them any time during the 8 days of study participation.

On the basis of their scope, surveys were also assigned a predetermined expiry mechanism, and before expiration, a survey could be accessed from the smartphone’s notification menu. The SMART survey was deployed on day 1 and was not set to expire until the citizen scientists completed or exited the survey. The first EMA (daily PA and contexts) was deployed every evening from day 1 to day 8 from 8 PM to 11:30 PM and was set to expire at midnight each day. The second EMA (barriers and facilitators of active living) was deployed using a combination of time (every day from 12 PM to 1 PM) and user-triggers and did not expire until completed or exited. The feedback surveys that collected paradata were time-triggered and were set not to expire until completed or exited. All daily time-triggered surveys were released as single-triggers without retriggering (ie, reprompting) the same surveys on the same day.

### Weather Data

To match the 8-day time period of each cycle of data collection, including the pilot data collection cycle, detailed hourly weather data (ie, daily maximum and minimum temperature, wind speed, precipitation, etc) will be obtained retrospectively from Environment Canada at the study locations [48-50].

### Analytical Strategies

The SMART Study’s comprehensive surveillance platform generates complex data that requires effective analytical strategies for linkage, validation, and modeling. Although the following strategy categories summarize some key approaches, the overall analyses are not limited to these examples.

#### Data Linkages

Given the diversity of data collected, linkages would be necessary before analyzing data. The following are examples of data linkages:

- Objective sensor data will be extracted in a cross-linked fashion (eg, GPS-accelerometer-screen state)
- Objective sensor data will be linked with subjective surveys and EMAs (eg, accelerometer-IPAQ)

#### Validation Analyses

Validation is a necessary step for cross-referencing the accuracy of subjective data with objectively collected measures. Validation analyses would include:

- Validation of smartphone deployed IPAQ with smartphone accelerometers
- Validation of IPAQ with EMAs

#### Cross-Sectional and Longitudinal Analyses

A variety of cross-sectional and longitudinal analyses would be conducted, including, but not limited to:

- Quantitative geo-coded mapping of barriers and facilitators of active living in different seasons
- Quantitative hierarchical influence of changing physical contexts on PA, and in turn, the influence of changing PA on eudaimonic well-being

#### Paradata Analyses

Objective (Wi-Fi) and subjective (feedback surveys) paradata will be used to adapt study implementation strategies temporally and survey triggering mechanisms within each 8-day data collection cycle. These paradata-informed strategies and mechanisms will play an important role in enhancing the rigor of study methods and address nonresponse, compliance, and attrition [62,63].

#### Hybrid Simulation Modeling

The sensor-based “big data” collected in SMART Study enables systems sciences approaches in developing hybrid simulation models using longitudinal data to inform decision makers at multiple levels [64-66]. For instance, simulation modeling can be conducted to test research questions that could inform policy makers of the impact of potential future active living interventions [67].

#### Pilot Data Analysis

The purpose of preliminary pilot data analysis is to depict evidence of successful implementation, as well as the flexibility of the SMART platform in which early identification of limitations could be addressed in subsequent data collection cycles. Initial pilot data analyses presented are examples of implementation and modifications and were conducted using SPSS version 14 (IBM Corp) software to understand the distribution of data and compliance for different surveys. Thematic analyses were also conducted using geo-coded photographs and audio files to understand major active living barriers and facilitators.

#### Data and Risk Management

To ensure confidentiality, data are encrypted before being stored on the smartphone and streamed to servers when a device establishes Wi-Fi connection. Any identifiable artefacts (eg, photos) are removed or deidentified before data analysis. Permissions built into the Ethica app are restricted so that the app cannot access personally identifiable information that is present on the smartphones (eg, contact list or network sites visited). MAC address anonymization is used to protect citizen scientists’ data based on a simple hash algorithm that is infeasible to backtrack. Risks and privacy management options are made clear to citizen scientists while obtaining informed consent. For example, citizen scientists can use the “snooze” function in the app that allows them to disable monitoring for a set duration.

## Results

Of the 317 citizen scientists (mean: 34.14 years; range: 18-69 years) registered for the spring 2017 pilot data collection cycle of SMART Study, only 5.0% (16/317) dropped out. Within the remaining 301 citizen scientists, 36.8% (112/301) were male, 60.1% (181/301) were female, 2.6% (8/301) were missing values; 54.1% (163/301) citizen scientists completed the 209-item SMART survey. Among those who completed the SMART survey, the proportion of females (65.6%, 107/163) was significantly higher (*P*<.001) than the proportion of males (34.3%, 56/163).

Overall, during the spring cycle, 57.4%, (173/301) citizen scientists responded (at least once) to the first EMA (Figure 6), and 21.5% (65/301) citizen scientists responded (at least once) to the second EMA (Figure 7), both of which were time-triggered. A total of 27.2% (82/301) citizen scientists initiated and completed the user-trigged second EMA at least once (Figure 7).

**Figure 9:**
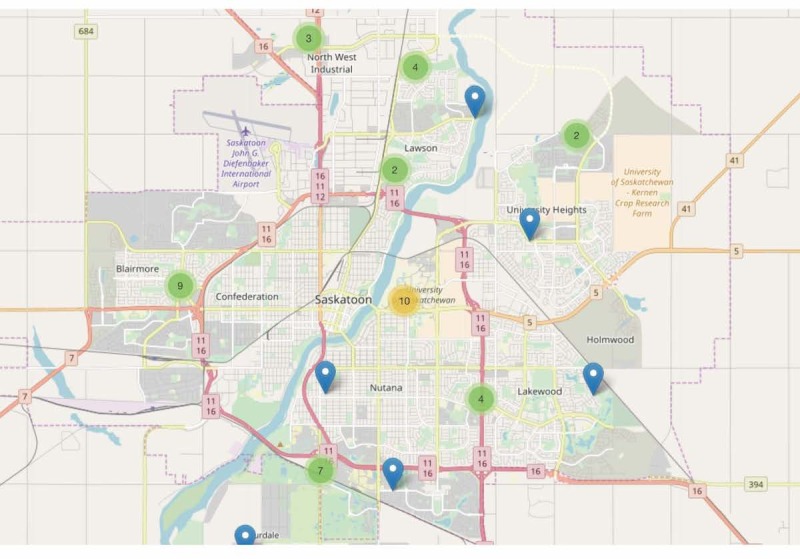
Geo-coded barriers and facilitators of active living in Saskatoon, *Saskatchewan, let’s move and map our activity* (SMART) Study.

**Figure 10:**
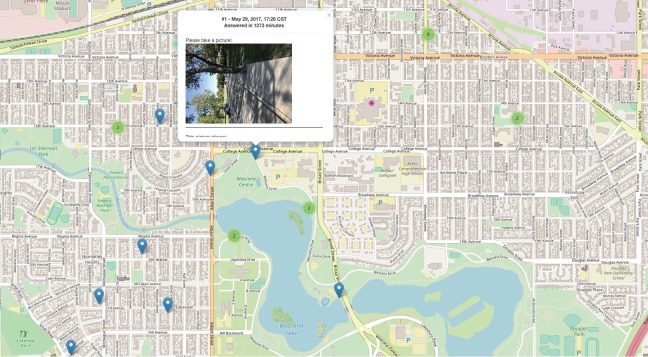
Geo-coded barriers and facilitators of active living in Regina, *Saskatchewan, let’s move and map our activity* (SMART) Study.

Figures 9 and 10 show examples of different resolutions of geo-coded pictures of barriers and facilitators of active living, as perceived by citizen scientists across Saskatoon and Regina. Further examination of these pictures revealed several patterns of perceptions of barriers and facilitators of active living, which were similar across both cities. These patterns have been summarized as “Community Voices” on the SMART Study website, as part of the integrated knowledge translation platform.

**Figure 11:**
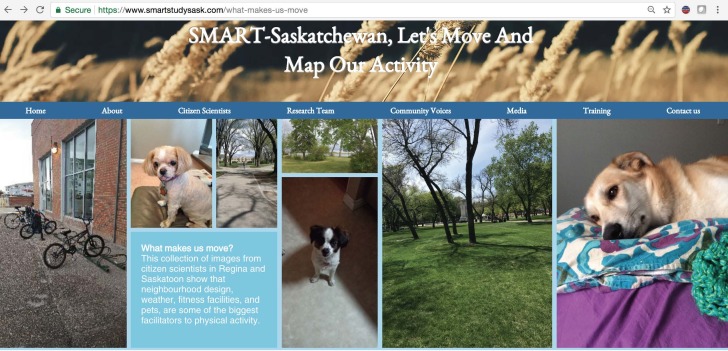
Community voices: pictures taken by the citizen scientists to illustrate their perception of active living facilitators.

In the “What makes us move in spring?” page of the SMART Study website [68], citizen scientists’ perceptions of active living facilitators were analyzed by common themes and summarized through pictures and the explanations that accompanied them. A selection of images that are representative of the larger sample of pictures taken by the citizen scientists show that neighborhood design enables active living, and access to fitness facilities, pleasant weather, and ownership of pets are perceived as facilitating active living (Figure 11).

**Figure 12:**
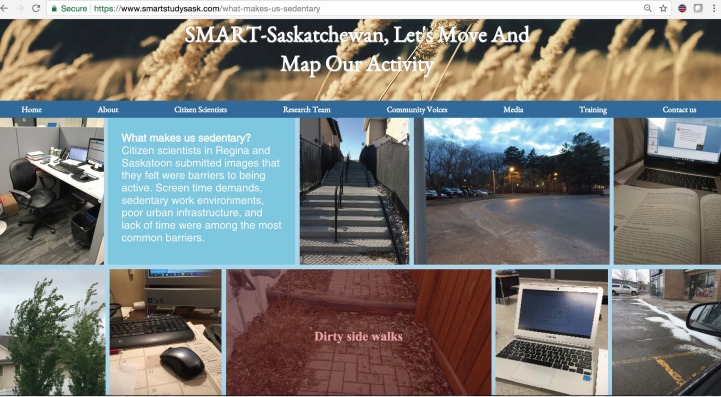
Community voices: pictures taken by citizen scientists to illustrate their perception of active living barriers.

Similarly, in the “What makes us sedentary in spring?” page of the SMART Study website [69], citizen scientists’ perception of active living barriers are summarized. Screen time demands, sedentary work environments, and poor urban infrastructure were revealed as the primary perceived barriers to active living (Figure 12). Apart from the physical and social contexts of active living depicted through citizen scientists’ pictures, the audio files provided another layer of evidence.

**Figure 13:**
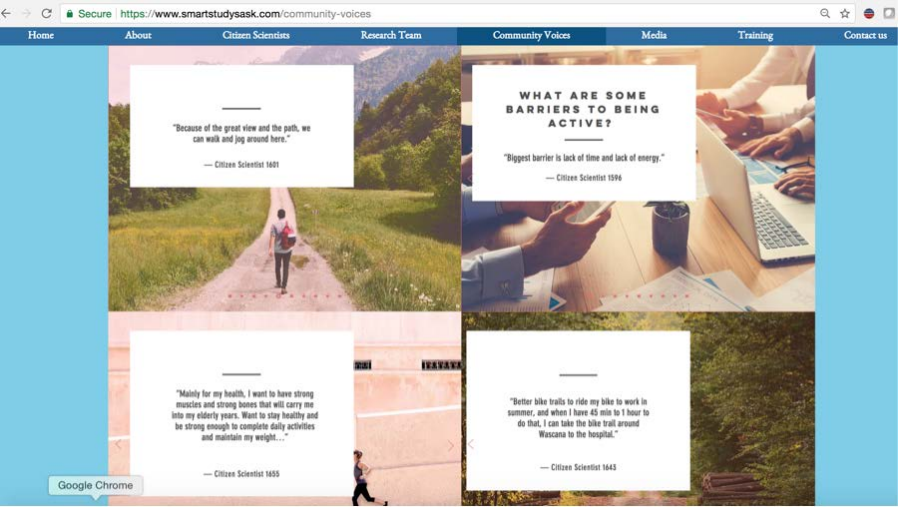
Community voices: citizen scientists’ perspective of active living facilitators and barriers as explained through their transcribed audio files.

In the “citizen scientist quotes” page of the SMART Study website, a representation of the larger sample of transcribed audio files portrays both individual and environmental levels of active living facilitators and barriers. At the individual level, citizen scientists with intrinsic motivations predominantly stated that maintenance of health is the main motivation to stay active. Another dominant individual level factor was the lack of time in one’s daily routine. At the environmental level, citizen scientists reported that key facilitators of active living included access to better biking and walking infrastructure, as well as aesthetics of the urban infrastructure (Figure 13).

## Discussion

### Principal Findings

The objective of the SMART Study was to develop a mobile and citizen science methodological platform for active living surveillance, knowledge translation, and policy interventions. Longitudinal surveillance of disparate behavioral data to inform policy interventions would benefit from citizen science, as evidence that carries the weight of public perception may provide impetus for decision makers to engage more effectively with researchers [34,35]. In engaging citizens, especially with respect to ALR, leveraging citizen-owned smartphones would be critical because of the ubiquitous presence of these devices, as well as the potential to repurpose smartphone sensors to obtain objective data [37,53].

To illustrate the evidence of successful implementation and flexibility of the SMART platform, preliminary pilot data analyses were conducted. The results of these analyses provide examples of how limitations could be identified and addressed between data collection cycles and how knowledge could be continuously translated as shown by the “Community Voices” pages of the study website.

### Strengths and Limitations: Lessons From Pilot Data Collection

The major strengths of the SMART Study platform include innovative approaches to capture complex data, ability to translate knowledge continuously, and flexibility to adapt the platform longitudinally from one seasonal data collection cycle to the next. For example, the limitations identified in the pilot data collection will be addressed in the next seasonal data collection, and this strategy will be repeated in the future.

The pilot data collection revealed that the main limitation is low compliance to both the 209-item SMART survey and the EMAs. Another apparent limitation was the higher proportion of females in the sample. Apart from these study-specific limitations that were identified at the pilot stage, key issues that need to be addressed are the differences between sensors across different smartphone models and validation of smartphone sensors with traditionally used active living measurement tools (eg, accelerometers and GPS loggers)

#### Compliance

During the pilot, citizen science compliance to stay in the study and provide continuous objective sensor data was very high (94.9, 301/317), with only 16 out of 317 registered citizen scientists opting to drop out. To our knowledge, the primary 209-item SMART survey is one of the most rigorous smartphone–based survey tools that integrates validated questionnaires capturing PA, SB, perception of indoor and outdoor environment, motivation, and eudaimonic well-being. Despite the considerable burden, 54.1% (163/301) citizen scientists completed the SMART survey in full. Nevertheless, 54.1% (163/301) completion rate would be construed as low compliance by traditional measures. The pilot was built to test the response rate with maximum burden. In the upcoming cycles of data collection, a combination of recruitment and deployment strategies will be employed (ie, administering the survey in segments over the 8 days) and compared within subsamples of the total sample to generate evidence for the most compliant approach for the SMART survey.

Compliance to EMAs varied widely, from 57.4% (173/301; EMA 1) to 27.2% (82/301; user triggered EMA 2) and 21.5% (65/301; time-triggered EMA 2). Similar variation was observed in a recent study by Bedard et al (2017) that examined the feasibility of EMA among young adults (mean age: 18.3 years), where the EMA compliance ranged from 64% to 47% [70].

However, a few key differences exist between other PA studies that used EMAs and the SMART Study [70-73]. First, the SMART Study age range was wider (18-69 years), with the mean age being 34.14 years. Second, the EMAs were different in content, context, length, and triggering mechanisms (user-triggered or time-triggered). More importantly, all time-triggered EMAs were triggered only once, whereas other studies used multiple prompts [70-73]. In the future data collection cycles, subsamples within the total sample of citizen scientists will be exposed to different frequency and timing of triggers to identify the optimum type, timing, and frequency of EMA triggers.

#### Citizen Scientist Retention

Several tactics will be used to reduce participant attrition. First, new citizen scientists will be recruited during each data collection cycle to maintain the proposed participant pool. Second, citizen scientists will be engaged through integrated knowledge translation processes to shape the course of the study [36]. Third, paradata will be used to address attrition by making the necessary changes to minimize burden. Finally, a constant feedback mechanism will be employed through social media (Facebook and Twitter) and the study website (ie, Community Voices), which will serve as a motivator for participants to stay engaged in the study on a long-term basis.

### Mobile Phone Surveillance via Citizen Science

The use of mobile phones is a relatively new phenomenon in ALR, and a recent systematic review conducted by Bort-Roig et al (2014) on the viability of smartphones to measure PA concluded that few studies have addressed the validity of smartphone measures [74]. Nevertheless, this review also points toward the potential of smartphones to increase participant engagement, with a call for future longitudinal studies to explore the measurement capabilities of smartphones [74].

Another recent review that appraised the technological features of smartphone apps promoting PA, highlights the versatility of smartphones for engaging participants through a combination of social networking–related extrinsic motivation and PA self-monitoring mechanism [75]. ALR using citizen science is now slowly emerging, albeit with smaller participant samples, as depicted by the deployment of a mobile app called the Stanford Healthy Neighborhood Discovery Tool [76]. In this study, 20 citizen scientists used an innovative app with minimal training to conduct a neighborhood environmental assessment through GPS-tracked walking routes, photographs, audio narratives, and surveys. They ultimately used this data to advocate for healthier neighborhoods [76].

The SMART Study combines the methodological capabilities and participant engagement opportunities provided by smartphones with a citizen science participatory approach to create a platform for longitudinal surveillance. SMART Study employs a mixed-methods design that repurposes smartphone sensor data critical to not only capturing PA and SB, but also the physical and social contexts within which these activities occur. For instance, the objective sensor data include GPS, accelerometer, and pedometer data that enable the categorization of physical and social contexts of PA; GPS-equipped screen state and gyroscope or magnetometer data that objectively capture the location of screen time and SB accumulation; and Bluetooth-Wi-Fi-Battery data that help confirm indoor location, as well as usage and compliance levels.

Moreover, SMART Study uses a combination of validated surveys, camera and audio-enabled EMAs, and feedback surveys (paradata) to not only triangulate the objective data with subjective and qualitative perception of citizen scientists, but also to take into consideration citizen scientists’ input in enhancing the surveillance platform on an ongoing basis. More importantly, this study provides citizen scientists an opportunity to proactively interact with the researchers in real time. Real-time data collection can be executed both via citizen scientist-triggered prompts and researcher-triggered prompts. For example, a citizen scientist can trigger a survey or upload a picture or audio file to provide important information about a certain barrier to active living. The platform could be adapted by automatically triggering a series of surveys in quick succession after receiving initial citizen scientist-triggered information to obtain more detailed data from the citizen scientists.

Another important aspect of SMART Study’s surveillance would be capturing seasonality’s influence on active living. On the basis of existing evidence, it is expected that warmer weather will be correlated with higher PA and lower SB [19-22]. Nevertheless, as PA and SB have been significantly influenced by wind speed and precipitation during a single seasonal transition in the participating cities [24,25], the influence of these weather factors on active living in different seasons would potentially provide new evidence for future behavioral and infrastructure-related interventions. Finally, in connecting the upstream determinants of active living with downstream outcomes of active living, SMART Study also obtains data on health outcomes, including eudaimonic well-being, self-rated health, and weight status.

### Platform for Integrated Knowledge Translation and Policy Interventions

The key to the successful implementation of SMART Study was strong collaborations with decision makers at multiple levels during the conceptualization of the study. Decision makers at the provincial level (appropriate Ministries), local jurisdictional level (cities of Regina and Saskatoon), community level (Young Men’s Christian Associations in Regina and Saskatoon), and institutional level (universities of Regina and Saskatchewan) were consulted and invited to become stakeholders in the SMART Study. With the support of these varied stakeholders, especially citizen scientists, SMART Study has been developed to translate knowledge using innovative means and to inform policy interventions at multiple levels. Integrated knowledge translation [77] will be conducted through citizen scientist-driven community voices webpage of the study website, social media accounts of the study, direct consultations with the stakeholders, traditional measures (eg, reports and publications), and strategic annual knowledge translation symposia involving all stakeholders and interested citizen scientists, where the agenda will revolve around knowledge exchange, identification of active living challenges, and proposal of solutions [78].

### Implications Beyond Active Living Research

As depicted by a recent study that leveraged smartphone data from 717,527 people residing in 111 countries across the globe [79], ubiquitous tools can be used effectively to identify inequities in active living within and between countries and influence decision makers to address them via healthy public policies across different sectors (eg, urban planning and education). Apart from the potential to influence policy, the ability to repurpose smartphone sensors (eg, accelerometers) minimizes cost, allows large-scale recruitment and retention, and provides avenues to test new approaches in ALR, such as a citizen science. This methodology of combining smartphone–based methods with citizen science can be replicated in other fields of study to address issues related to health policy and beyond.

### Conclusions

SMART Study introduces a novel and replicable smartphone–based methodological platform for surveillance, knowledge translation, and policy intervention by using citizen science. The preliminary results show the flexibility of such a surveillance platform in identifying and addressing limitations based on empirical evidence. The results also show the successful implementation of a platform that engages participants to catalyze policy interventions. Although SMART Study is currently geared toward surveillance, using the same platform, active living interventions could be remotely implemented [74,80]. In conclusion, SMART Study is the first mobile, citizen science surveillance platform utilizing a rigorous, longitudinal, and mixed-methods investigation to temporally capture behavioral data for knowledge translation and policy interventions.

## Abbreviations

ALR: active living research
EMA: ecological momentary assessment
GPS: global positioning system
IPAQ: International Physical Activity Questionnaire
PA: physical activity
SB: sedentary behavior
